# Regional differences in the expression of K^+^–Cl^−^ 2 cotransporter in the developing rat cortex

**DOI:** 10.1007/s00429-013-0515-9

**Published:** 2013-02-19

**Authors:** Krisztina Kovács, Kaustuv Basu, Isabelle Rouiller, Attila Sík

**Affiliations:** 1 Neuroscience Networks Group, Neurobiology and Neuropharmacology, College of Medical and Dental Sciences, School of Clinical and Experimental Medicine, University of Birmingham, Birmingham, B15 2TT UK; 2 Department of Anatomy and Cell Biology, McGill University, Montreal, QC H3A 0C7 Canada

**Keywords:** Development, Intracellular chloride homeostasis, KCC2, Neocortex, Paleocortex, Spine genesis, Inhibition

## Abstract

The type 2 potassium–chloride cotransporter (KCC2) is the main regulator of intracellular chloride concentration in CNS neurons, and plays a crucial role in spine development that is independent of its ion cotransport function. The expression pattern of KCC2 is upregulated during postnatal development showing area and layer-specific differences in distinct brain areas. We examined the regional and ultrastructural localisation of KCC2 in various areas of developing neocortex and paleocortex during the first two postnatal weeks. Light-microscopy examination revealed diffuse neuropil and discrete funnel-shaped dendritic labelling in the piriform and entorhinal cortices at birth. Subsequently, during the beginning of the first postnatal week, diffuse KCC2 labelling gradually started to appear in the superficial layers of the neocortex while the punctate-like labelling of dendrites in the piriform, entorhinal and perirhinal cortices become more pronounced. By the end of the first postnatal week, discrete dendritic expression of KCC2 was visible in all neocortical and paleocortical areas. The expression level did not change during the second postnatal week suggesting that, in contrast to hippocampus, adult pattern of KCC2 in the cortical cells is already established by the end of the first postnatal week. Quantitative electron microscopy examination revealed that in superficial layers of both neo- and paleocortex, the majority of KCC2 signal was plasma membrane associated but the number of transport vesicle-associated immunosignal increased with development. In deep layers, KCC2 immunolabeling was evenly distributed in plasma membrane and transport vesicles showing no obvious change with maturation. The number of KCC2 immunogold particles increased in dendritic spines with no association with synapses. This observation points to the dual role of KCC2 in spine genesis and ion cotransport.

## Introduction

Fast hyperpolarizing inhibition in the central nervous system (CNS) is mediated by ligand-gated anion channels (i.e. GABA_A_ and glycine receptors) that gate currents predominantly carried by Cl^−^ and to a lesser extent to HCO_3_^−^ (Farrant and Kaila 2007; Kaila 1994). The action of GABA_A_ and glycine receptors highly depends on the intracellular chloride concentration ([Cl^−^]_i_). In the adult brain, the vast majority of the neurons has low [Cl^−^]_i_ resulting in Cl^−^ influx during channel opening and subsequent hyperpolarization of the cell (Eccles 1966; Kaila 1994). In several regions of immature brain, such as hippocampus, auditory cortex, cerebellum, inferior olive or in pathological conditions (Owens et al. 1996; Cohen et al. 2002; Kahle et al. 2008; Papp et al. 2008; Blaesse et al. 2009), due to the high [Cl^−^]_i_, the activation of GABA_A_- or glycine receptor causes an outward flux of Cl^−^ that depolarizes the cell (Cherubini et al. 1991; Ehrlich et al. 1999; Zhang et al. 1990). Maintaining the resting Cl^−^ equilibrium potential more negative than the resting membrane potential requires a Cl^−^ extrusion mechanisms in the postsynaptic cell (Thompson and Gahwiler 1989). Cation-chloride co-transporters including Na^+^–K^+^–2Cl^−^ co-transporters (NKCC) and K^+^–Cl^−^ co-transporters (KCC) play a key role in the regulation of [Cl^−^]_i_ in the nervous system (Hiki et al. 1999; Race et al. 1999; Delpire and Mount 2002; Payne et al. 2003; Mercado et al. 2004). Potassium–chloride co-transporter 2 (KCC2), found exclusively in neuronal cells, is responsible for the [Cl^−^]_i_ homeostasis by constantly transporting K^+^ and Cl^−^ out of the cell (Rivera et al. 1999; Payne et al. 1996; Lu et al. 1999; DeFazio et al. 2000).

During neuronal development, [Cl^−^]_i_ decreases significantly from embryonic to early postnatal life. Several lines of evidences show GABA_A_-mediated excitation in hippocampal pyramidal neurons during the first postnatal week (Cherubini et al. 1991; Ben-Ari et al. 1989; Gulyas et al. 2001) that disappears around the beginning of second postnatal week. The ontogenetic change of GABA_A_-receptor responses resulting from the decrease of [Cl^−^]_i_ is attributed to the developmental upregulation of KCC2 (Williams et al. 1999; Rivera et al. 1999; DeFazio et al. 2000). Although recent studies indicated that excitatory action of GABA is necessary for the proper maturation of the cortical neurons (Hubner et al. 2001; Stein et al. 2004; Cancedda et al. 2007), there is limited information about the cortical distribution of KCC2 during early postnatal development (Takayama and Inoue 2010).

KCC2 plays an important role in the development of dendritic spines, and synaptogenesis and premature expression of KCC2 induces spine density increase on cortical neurons (Fiumelli et al. 2012; Cancedda et al. 2007; Horn et al. 2010). It was recently hypothesized that the interaction between KCC2 and the actin cytoskeleton plays an important role during activity dependent assembly of developing cortical circuits and have functional consequences for brain plasticity (Fiumelli et al. 2012). Because of the dual function of KCC2 (ion cotransport independent synaptogenesis, and ion cotransport) during the perinatal period, it is important to have knowledge about the expression pattern of KCC2 in early postnatal period in the cortex.

In this study, we examined the expression of KCC2 in different cortical regions of developing rat brains and compared to the hippocampal KCC2 expression pattern using light and electron microscopic analysis. Since previous studies (i.e. Rivera et al. 1999) showed that by the second week of postnatal life KCC2 expression reaches maximum level, our study was limited to this period.

## Materials and methods

### Animals

Twenty young (postnatal days P0, P2–P6, P12–15; 2 animals per age studied) Wistar rats were either deeply anaesthetised with chlornembutal (0.3 mL/100 g body weight) or by cooling and then perfused intracardially with 0.9 % saline followed by 4 % paraformaldehyde, and 0.05 % glutaraldehyde dissolved in phosphate buffer (PB, pH = 7.2, 0.1 M). After fixation, 60 μm thick coronal sections were cut using a Vibratome. Following extensive washes in PB, the sections were immersed in a mixture of 25 % sucrose and 10 % glycerol in 0.1 M PB, and freeze-thawed in liquid nitrogen to increase the penetration of antisera during immunostaining. All experiments were carried out according to the National Institute of Health guiding principles.

### Immunoperoxidase reactions for KCC2

Sections were washed three times for 30 min between each step in 50 mM Tris buffer saline (TBS, pH = 7.4) and blocked for 45 min with 2 % w/v bovine serum albumin (BSA, Sigma-Aldrich) in TBS. Thereafter, they were incubated for 2 days at 4 °C with the primary antibody directed against KCC2 (rabbit anti-KCC2 antiserum, 1: 500) (Williams et al. 1999; Gulyas et al. 2001) then with secondary antibody (biotinylated goat anti-rabbit IgG, 1:300, 4 h, Vector Laboratories) in TBS followed by incubation with Avidin–Biotin Complex (ABC, 1:400, Vector Laboratories) for 3 h. After several washes in TBS, the immunoperoxidase reaction was carried out using 3′3-diaminobenzidine tetrahydrochloride (DAB, Fluka Sigma-Aldrich, 0.05 % w/v in Tris) intensified with nickel (Wouterlood 1988) as a chromogen and 0.01 % v/v H_2_O_2_ as oxidant. Sections were treated with 1 % OsO_4_ for 1 h, dehydrated in graded ethanol (70 % v/v ethanol containing 1 % w/v uranyl acetate) and in propylene-oxide and embedded in Durcupan (Fluka Sigma-Aldrich).

### Pre-embedding immunostaining for KCC2

To reveal the subcellular distribution of KCC2, after the primary antiserum, sections were incubated in TBS containing goat anti-rabbit IgG coupled to 1 nm gold particles (Amersham, UK) diluted 1:50 overnight at 4 °C. Then, tissues were washed with PB, fixed in 1 % v/v glutaraldehyde solution for 10 min, and washed three times 10 min in Enhancement Conditioning Solution (Aurion Immunoresearch, Wageningen, The Netherlands). Finally, the gold particles were intensified with R-Gent silver intensification solution (Aurion). At the end of the immunogold reactions, sections were incubated in 0.5 % v/v O_s_O_4_ for 30 min at 4 °C and dehydrated and embedded as above. Regions of interest were re-embedded and sectioned serially in 50 nm sections. Ultrathin serial sections were collected on formvar-coated single slit copper grids, counterstained with lead citrate.

To reveal the relative distribution of KCC2, samples were taken from entorhinal, piriform and somatosensory cortices. The subcellular localization of the immunogold particles was examined in ultrathin sections cut from the surface (max 2 μm) of the pre-embedding immunostained light microscopic sections.

Electron micrographs were taken using a Tecnai 12 electron microscope equipped with Gatan CCD camera. Sections containing DAB labelling were analysed with an Olympus BX61 microscope equipped with an EXi Blue (QImaging, UK) digital camera. Images were analysed using Image Pro 7 (Media Cybernetics, USA) software.

## Results

### Distribution of KCC2 protein in the cortex

**Fig. 1:**
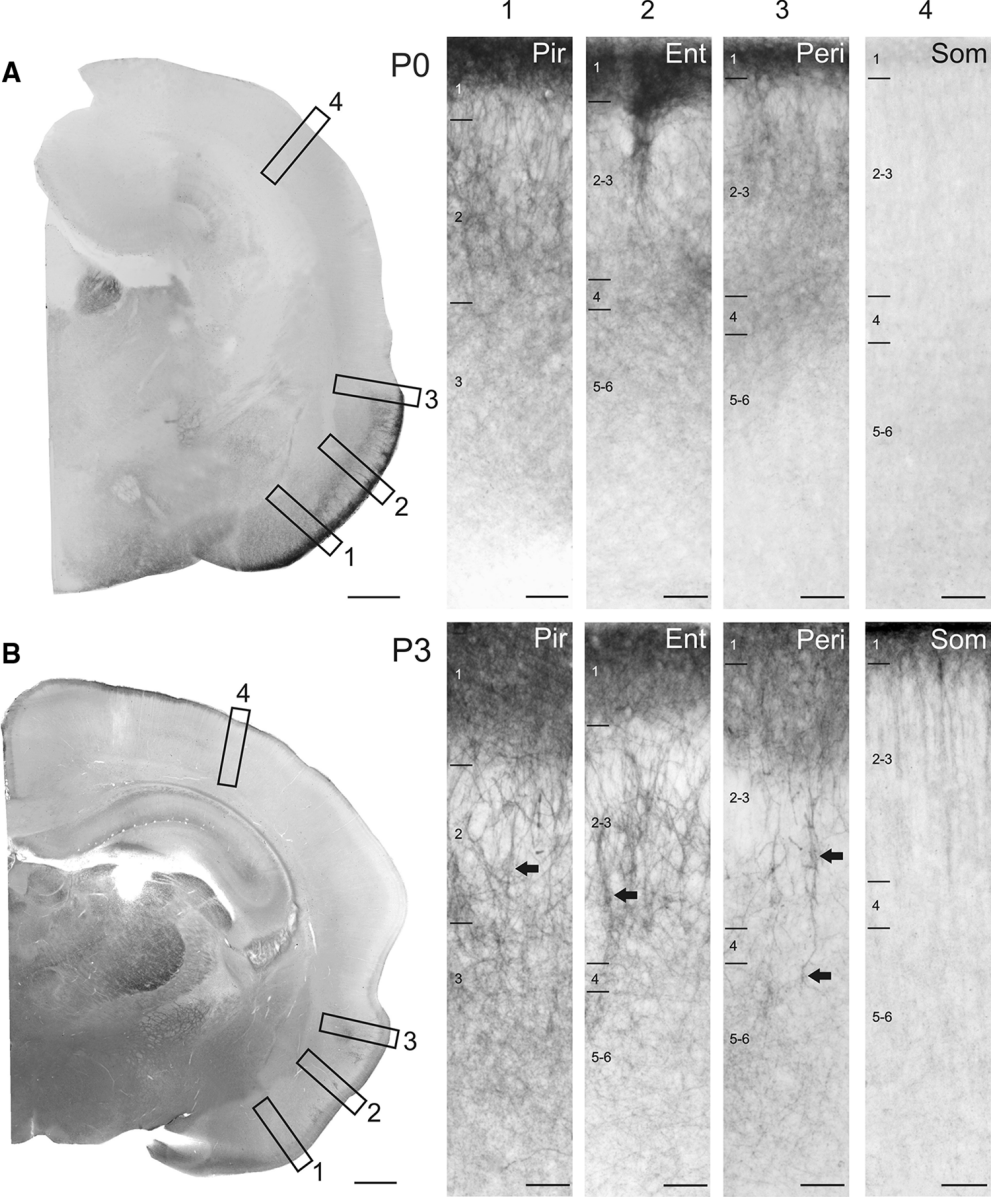
Developmental expression of KCC2 in the paleo- and neocortex at *P0* and *P3*. **a** Strong dendritic labelling is visible already in *P0* in the piriform (*Pir, a1*), entorhinal (*Ent, a2*) and perirhinal (*Peri, a3*) cortices, especially in the superficial layers. In the somatosensory cortex (*Som, a4*), only a faint and diffuse immunoreaction could be observed. **b** At *P3*, the distribution of KCC2 immunosignal is similar in the piriform (*b1*), entorhinal (*b2*) and perirhinal (*b3*) cortical areas, although higher level of signal can be observed in deeper layers (3–6) than at *P0*. Immunostaining starts to appear in the superficial layers of the neocortical regions (**b**) such as somatosensory cortex (*b4*). Elongated pyramidal shaped somata and thick apical dendrites are indicated by *arrows*. *Scale bars*: **a**, **b**: 500 μm;* a1–a4*: 50 μm;* b1–b4*: 50 μm

**Fig. 2:**
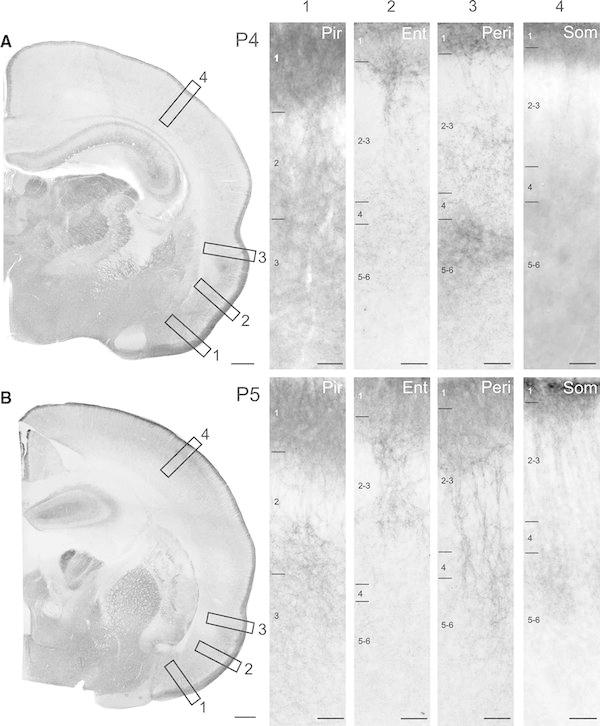
Developmental expression of KCC2 in the paleo- and neocortex at *P4* and *P5*. **a** At *P4*, both the paleo- and neocortex show distinct KCC expression. Signal intensity increases in the piriform (*a1*), entorhinal (*a2*) and perirhinal cortices (*a3*) especially in the layer 3–6. Immunolabelling is more homogeneous in the somatosensory area (*a4*) but strong KCC2 expression is present in the layer 1. Note the strongly labelled neuronal groups on the* a1–a3* but not in* a4*. **b** At *P5* signal shows similar intensities in the piriform (*b1*), entorhinal (*b2*) and perirhinal (*b3*) cortices, while in the somatosensory cortex immunosignal increases (*b4*) and funnel-shaped apical dendrite labelling becomes more pronounced. *Ent* entorhinal cortex, *Peri* perirhinal cortex, *Pir* pirform cortex, *Som* somatosensory cortex. *Scale bars*: **a**, **b**: 500 μm,* a1–a4*: 50 μm;* b1–b4*: 50 μm

**Fig. 3:**
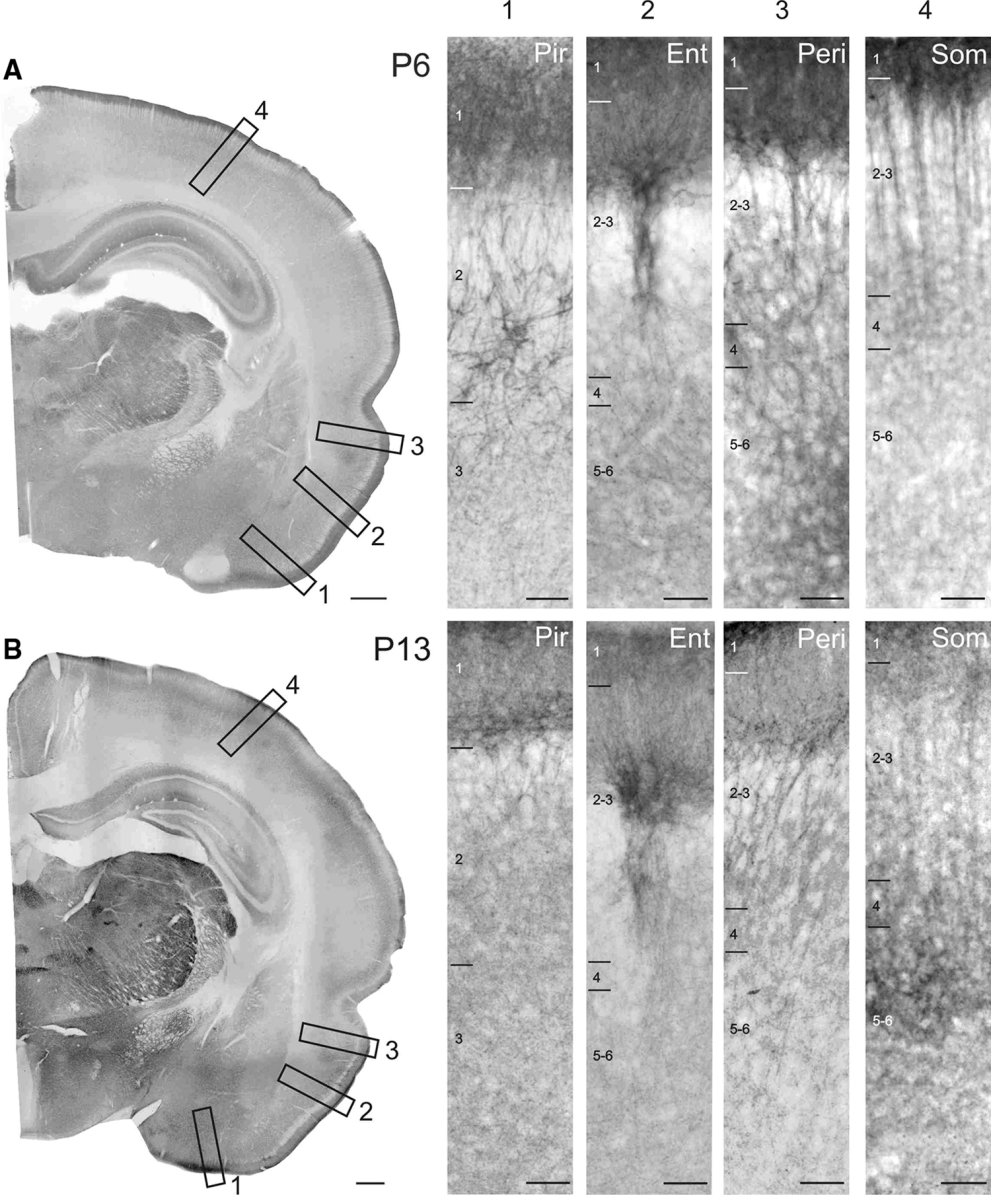
Developmental expression of KCC2 in the paleo- and neocortex at *P6* and *P13*. **a** Already at *P6*, the KCC2 labelling present in all cortical areas showing strong dendritic labelling in all cortical layers (*a1–a4*). **b** The signal intensity and KCC2 expression pattern did not change at *P13* (*b1–b4*). *Ent* entorhinal cortex, *Peri* perirhinal cortex, *Pir* pirform cortex, *Som* somatosensory cortex. *Scale bars* **a**, **b** 500 μm,* a1–a4* 50 μm;* b1–b4* 100 μm

To reveal the age-dependent changes in the KCC2 expression of neocortex and paleocortex, we investigated the expression pattern of KCC2 at different developmental stages (P0, P2–6 and P12–15). In newborn animals (P0), a diffuse neuropil staining and strong discrete dendritic labelling were present in the paleocortex already showing area- and layer-specific patterns (Fig. 1a). Extensive labelling was observed in the entorhinal and piriform cortices of somata and dendrites presumably belonging to pyramidal cells. The expression of KCC2 was the densest in layer 1 followed by the middle layers. In the entorhinal cortex, bundles of strongly labelled dendrites were visible that originated from the middle layers and gave rise to dens labelling of funnel-shaped dendritic arborisation in the superficial layers (Fig. 1a1–a3). In other cortical areas (e.g. somatosensory, auditory, motor cortices), KCC2 expression was absent except a faint neuropil labelling in the layer 1 (Fig. 1a4). At early developmental stage (P3), entorhinal and piriform cortices were heavily labelled (Fig. 2b). The most prominent expression of KCC2 was fund in the layer 1, where presumably of local inhibitory cells and apical dendrites of pyramidal cells concentrated. At this age, diffuse labelling in layer 1 started to appear in the perirhinal and entorhinal cortices along with somatosensory, auditory, motor cortices. Similarly to P0 animals, strongly labelled patches of dendritic tufts presumably belonging pyramidal cell were observed in the superficial layers of entorhinal and perirhinal cortices. Additionally, elongated pyramidal shaped somata and thick apical dendrites of layer 5 pyramidal cells were apparent (Fig. 1b1–b3). Basal dendrites arborizing mainly in the same layer around the cell body were also labelled extensively. Diffuse immunolabeling was also visible in layer 6. In the piriform cortex, dense dendritic labelling in the superficial layers was observed; however, it was considerably more homogenous lacking the patchy appearance which has been seen in the entorhinal cortex both at P0 and P3. At P4, the strong diffuse immunostaining of layer 1 was already present in almost all cortical areas (Fig. 2a). In sensory (somatosensory, auditory) and motor cortices, the dens labelling was divided by vertically running bundles of apical dendrites and their funnel-shaped dendritic arborisations (Fig. 2a4). Paleocortex (entorhinal and piriform cortices) showed similar intensive dendritic and somatic labelling as at P3 (Fig. 2a1–a3). At P5, discrete Golgi-like dendritic and somatic labelling of large number of neurons was visible in entorhinal and piriform cortices without diffuse neuropil staining (Fig. 2b1–b3). In somatosensory and motor cortices, the bundles of pyramidal cell dendrites are more pronounced in layers 2/3 and layer 1. Additionally, a faint diffuse staining was observed in the supragranular and infragranular layers presumably belonging to the basal dendrites of pyramidal cells (Fig. 2b4). At the end of the first postnatal week, around P6, the cortical expression of KCC2 reached the overall staining pattern observed in P14 animals (Fig. 3a). At P13, expression became more diffuse in all layers of the neo- and paleocortex (Fig. 3b).

### Ultrastructural localisation of KCC2 in the developing cortex

**Fig. 4:**
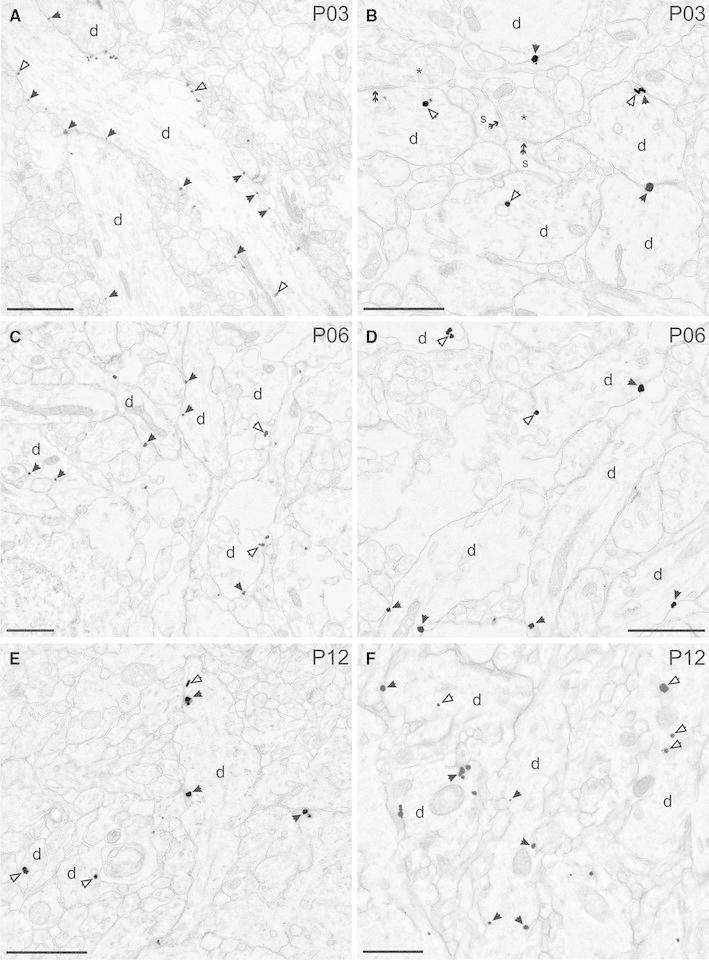
Ultrastructural localisation of KCC2 in the developing cortex. **a**, **b** Electron micrographs showing the distribution of immunogold particles in the entorhinal cortex at *P3*. Silver-intensified gold particles are frequently observed in the dendritic plasma membrane (*black arrows*) and in transport vesicles (*open arrows*). Note that KCC2 labelling is not associated to pre- or postsynaptic membranes (*double arrow*). **c**, **d** Subcellular localisation of KCC2 in dendritic plasma membranes of dendritic shafts and transport vesicles in the somatosensory cortex at *P06*. **e, f** At P12, in the piriform cortex immunogold particles were predominantly found in dendritic plasma membranes and transport vesicles but not around synapses. *d* dendrite, *s* spine, *asterisk* presynaptic terminal. *Scale bars* **a** 2 μm, **b–f** 1 μm

**Fig. 5:**
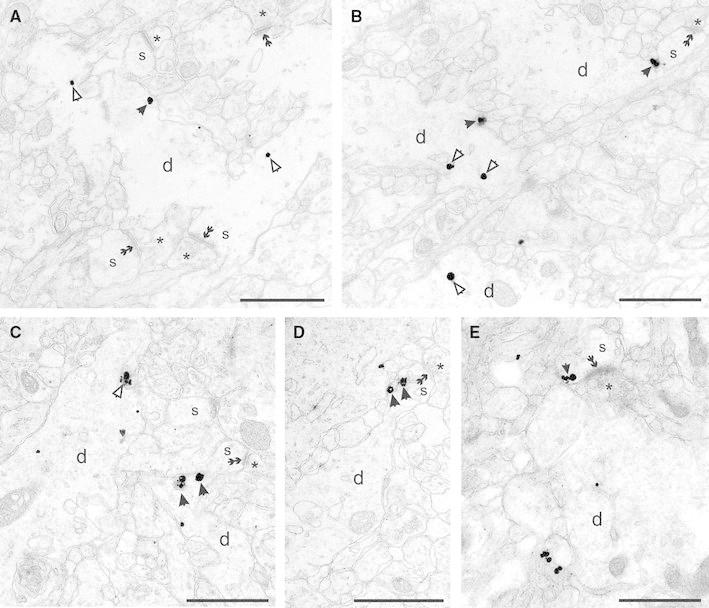
KCC2 is not localised in spine heads in the cortex of *P12* rat. **a** Electron micrograph showing the distribution of immunogold particles in dendritic plasma membrane (*black arrows*) and in transport vesicles (*open arrows*). Dendritic spines (*s*) receiving excitatory synapses (*double arrowheads*) are unlabelled. Presynaptic terminals are depicted by *asterisks*. **b–d** When immunogold signal was observed in spines it was found in spine necks associated with endoplasmic reticulum (*arrowheads*). *Open arrowheads* depict gold particles in transport vesicles. **e** In very rare occasions, silver-intensified gold particle was observed in spine head. *d* dendrite, *s* spine, *asterisk* presynaptic terminal. *Scale bars* 1 μm

The presence of KCC2 in neocortical cells has already been reported in early postnatal life. Low to moderate level of signal has been detected by in situ hybridisation from P0 rat with further developmental increase up to P14 (Clayton et al. 1998). The aforementioned technique is able to detect the gene expression of KCC2 in the tissue, but provides no information about protein expression and precise subcellular localisation of the protein. Therefore, in the next part of our study, we focused on the cell-surface distribution of KCC2 protein using immunogold electron-microscopy technique. Since the expression pattern of KCC2 is different in the neocortex and paleocortex, and the superficial layer labelling showed marked difference to deep layers in light microscope, we investigated the subcellular distribution of KCC2 in these areas. In the paleocortex, at early postnatal age P2 immunogold particles were localised predominantly in the dendritic plasma membrane; however, they were also associated frequently with membranes of transport vesicles (Fig. 4). At P6, silver-intensified immunogold particles were found in the dendritic plasma membrane and the inner membrane structures. Similarly to P3, there was no apparent accumulation in the vicinity of excitatory or inhibitory synapses. After the first postnatal week, the ultrastructural localisation of the KCC2 gold particles was qualitatively similar to the pattern at P12. The immunogold particles were found in the plasma membrane of dendrites and rarely of somata, as well as occurred on transport membranes. In the neocortex, we observed immunogold distribution that is similar to the paleocortex. The majority of gold particles was associated with plasma membrane, but numerous gold signals were observed on transport vesicles. Although immunogold was occasionally found in dendritic spines, no obvious association with synapses could be determined. Interestingly, silver-intensified gold particles depicting the subcellular expression of KCC2 protein was found very rarely in the spine heads (Fig. 5e). As mentioned above, KCC2 was typically observed in transport vesicles or in the plasma membrane, but occasionally was seen close to dendritic spines (Fig. 5a). At P12, when the number of spines is substantial, immunogold signal was localised in the spine neck where associated with endoplasmic reticulum (Fig. 5b–d).

**Fig. 6:**
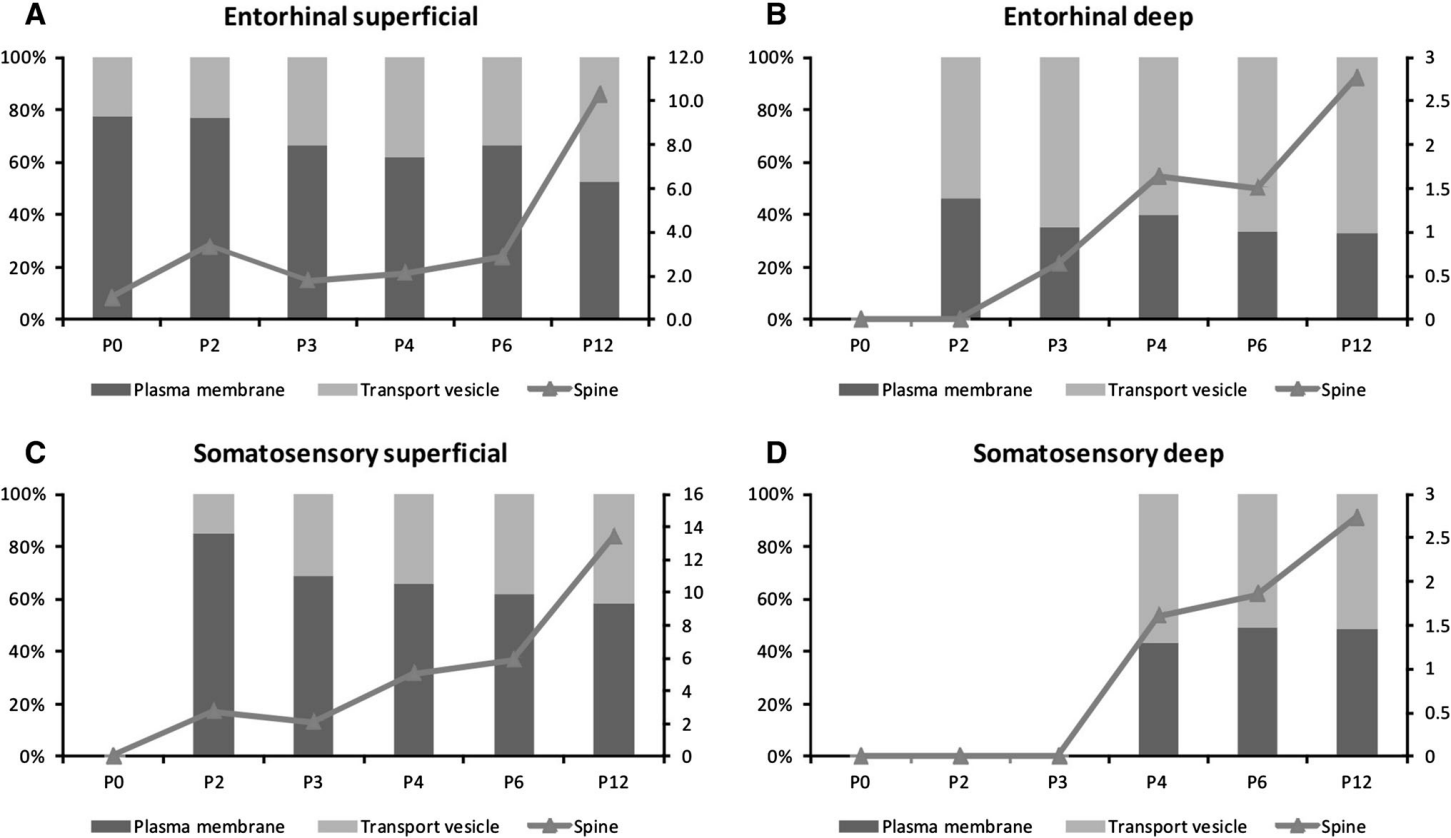
Quantitative analysis of the subcellular distribution of KCC2 immunosignal in the paleocortex and neocortex. In the superficial layer of both entorhinal (**a**) and somatosensory (**c**) cortex, the number of transport vesicle-associated immunogold particles increase with maturation. In deep layers (**b**, **d**) we did not find similar increase. In all layers and areas the number of immunogold particles in dendritic spines increased with age.* Right* *Y* axes of graphs indicate the percentage of immunogold particles in spines

In order to gain better insight to the possible function of KCC2, we quantified the distribution of the immunogold signal in the paleo- and neocortex (Table 1). In superficial layers of the entorhinal cortex (layer I and II), the majority of gold particles was found on the plasma membrane at P0, and only 22.8 % were associated with transport vesicles. With development this ratio changed and at P12 47.3 % of the gold particles was found on transport vesicles (Fig. 6a). Immunogold signal was rarely observed in dendritic spines at early age (1 % at P0) that increased to over 10 % at P12 (Fig. 6a; Table 1). In deep layers of the entorhinal cortex, the plasma membrane/transport vesicle-associated immunosignal ratio was different from the superficial layer. Although as in superficial layers, the number of transport vesicle-associated gold particles increased by development (P2: 53.5 %; P12: 66.8 %), in general, the gold signal was less frequently found on plasma membrane. In deep layers, even less gold particle was found in spines than in superficial layer and reached only 2.8 % at P12 (Fig. 6b; Table 1).

**Table 1:** Percentage and number of gold particles analysed in the quantitative EM study

	P0				P2				P3				P4				P6				P12			
	Ent		Som		Ent		Som		Ent		Som		Ent		Som		Ent		Som		Ent		Som	
	*S*	*D*	*S*	*D*	*S*	*D*	*S*	*D*	*S*	*D*	*S*	*D*	*S*	*D*	*S*	*D*	*S*	*D*	*S*	*D*	*S*	*D*	*S*	*D*
Plasma membrane (%)	77.2	–	–	–	76.7	46.5	84.9	–	66.6	35	69	–	61.7	40.2	65.7	43.1	66.7	33.7	61.6	49	52.7	33.2	58.2	48.4
Transport vesicle (%)	22.8	–	–	–	23.3	53.5	15.1	–	33.4	65	31	–	38.3	59.8	34.3	56.9	33.3	66.3	38.4	51	47.3	66.8	41.8	51.6
Spine (%) and *n*	1 (1)	–	–	–	3.3 (4)	0	2.7 (2)	–	1.8 (6)	0.6 (1)	21 (6)	–	2.1 (3)	1.6 (2)	5.1 (5)	1.6 (5)	2.9 (9)	1.5 (5)	5.9 (19)	1.9 (8)	10.3 (17)	2.8 (6)	13.4 (26)	2.7 (10)
Number of particles	101	–	–	–	120	43	73	–	338	157	287	–	141	122	99	311	315	332	323	431	165	217	194	366

In the somatosensory cortex, KCC2 expression was virtually absent at P0 and P2 in deep layers, thus quantitative EM study was not performed on P0 samples, and in deep layers at P2 and P3. Similar to the paleocortex, plasma membrane labelling was dominant in the superficial layer at P3 (69 %) and with maturation the transport vesicle-associated labelling increased (P3: 31 %; P12: 41.8 %). In deep layers, the distribution of transport vesicle- and plasma membrane-associated immunogold particles were approximately the same (Fig. 6; Table 1). Similar to the paleocortex, the ratio of immunosignal in the spine increased with age reaching 13.4 % in the superficial layer. Whenever we found gold particles close to either asymmetric or symmetric synapse, we measured the distance to the edge of the synapse. The average distance varied between 0.13 and 0.47 μm with large standard deviation (0.07–0.33 μm).

## Discussion

In this study, we examined the cellular and subcellular expression pattern of KCC2 in the developing rat brain. Our light- and electron-microscopy immunohistochemical analyses revealed a distinct area specific distribution and an age-related increase of KCC2 protein. Intense KCC2 labelling was detected first in the basal part of the neocortex (piriform and entorhinal cortices). Already in newborn animals, strong dendritic and somatic labelling was evident in the superficial layers. During the development, the expression of KCC2 gradually increased and after the first postnatal week it reached the adult pattern. In contrast, in neocortical areas, immunostaining was absent in P0 animals and a diffuse labelling of layer 1 appeared only after P3. The signal intensity increased with age and funnel-shaped dendritic labelling originated presumably from layers 2–3 and 5 pyramidal cells became visible. Similarly to paleocortex, an age-related increase of protein expression was detectable and the signal intensity could not be differentiated from the adolescent (P15) by P6.

### KCC2 expression correlates with the development of the telencephalon

Our neuroanatomical observations are consistent with previous studies using in situ hybridisation, Western and Northern blot, ribonuclease protection analysis and electrophysiological techniques (Clayton et al. 1998; Rivera et al. 1999; Lu et al. 1999; Fukuda et al. 1998; DeFazio et al. 2000). Developmental studies pointed out that the ontogeny of KCC2 expression in the CNS is in accordance with neuronal maturation and follows a caudal-rostral pattern (Li et al. 2002; Stein et al. 2004). KCC2 protein was first detected in the postmitotic neurons of the spinal cord and subcortical neurons during embryogenesis and is then gradually increased in higher brain structures (Stein et al. 2004; Wang et al. 2002). Interestingly, at birth, adult protein levels were already established in the spinal cord and the brainstem. In cortical neurons, the expression of KCC2 begins prenatally and increases after birth (Stein et al. 2004; Wang et al. 2002). As a general rule, KCC2 mRNA could be detected as soon as a particular immature neuron had reached its final position in the cortex. In the cerebellum, KCC2 transcripts were already detected at E12, a developmental stage where the formation of axon extensions of cerebellar neurons occurs (Hatten et al. 1997). In the basal older parts of the cortex, such as the piriform cortex, neurons are generated between E13 and E16, in the same time when intense signals for KCC2 transcripts were also reported (Clayton et al. 1998). KCC2 expression increased in parallel to the differentiation of the cortical neurons. Paleocortical areas were characterised by a strong somatic and dendritic labelling in the superficial layers immediately after the birth at P0. Presumably apical dendritic tufts in the piriform, entorhinal cortices stained strongly for KCC2. Neocortical neurons start to differentiate later at E14 and finish neurogeneration around E20 (Bayer 1980, 1986). Faint KCC2 protein signal was already detected in Western blot analysis of the cortex at E15.5, which further increased at E18.5. However, at E18.5 mRNA expression was mainly restricted to piriform cortex, and a faint signal in the neocortical areas was visible first around P3 which was remarkably enhanced by P7 (Stein et al. 2004). We found that expression of KCC2 in the neocortex is consistent with previous immunohistochemical studies (Stein et al. 2004; Clayton et al. 1998; Wang et al. 2002; Takayama and Inoue 2010). Generally, a diffuse labelling started to appear at P3 and P4 in the sensory areas of rat neocortex. Similarly to previously reported, an increase of KCC2 expression was observed over the entire neocortex during further development and reached adult levels in the first postnatal week. These observations might explain why GABA_A_-receptor agonists are useful in neonatal brainstem epilepsies but control seizures in the rostral parts of the CNS only later in life.

Similarly to hippocampus, the neocortex also displays a developmental increase in the KCC2 protein level, although the later reaches the adult expression pattern already around the first postnatal week (Gulyas et al. 2001; Wang et al. 2002; Stein et al. 2004; Takayama and Inoue 2010). Interestingly, alterations in the expression pattern of KCC2 during development could occur between different species. KCC2 mRNA is present abundantly in guinea pig hippocampus already at embryonic day E42 and is not significantly upregulated during postnatal development (Rivera et al. 1999).

### Subcellular localisation of KCC2

Electron microscopic examination revealed that in the entorhinal cortex, KCC2 is localised not only in transport vesicles but also mainly in the dendritic plasma membranes at early developmental stage at P0. In contrast to hippocampus (Gulyas et al. 2001), where KCC2-immunoreactive transport vesicles were gradually decreased with the age, in the superficial layer of both neocortex and paleocortex, the number of transport vesicle-associated immunogold particles steadily increased. This finding suggests that in cortical cells, the KCC2 synthesis and transport to the plasma membrane are increased during development. Alternative explanation for the observation is the increase in KCC2 recycling in the dendritic plasma membrane. In the deep layers, we did not observe similar trend and found the majority of KCC2 immunosignal in transport vesicles possibly because in deep layers, we analysed more somata where many immunogold particles found inside the somatic cytoplasm and seldom on the plasma membrane.

In the hippocampus, KCC2 is highly expressed in the vicinity of excitatory synapses presumably close to extrasynaptic GABA_A_ receptors (Gulyas et al. 2001; Baldi et al. 2010). Additionally, in thalamic relay cells, KCC2 was also found in close association with asymmetric synapses formed by cortical afferents (Bartho et al. 2004). In contrast to previous observations, in the cortex, KCC2 does not seem to be associated with inhibitory or excitatory synapses. Although immunogold particles were observed in dendritic spines, we observed them in the spine apparatus or close to the spine neck most of the time. Although we found gold particles on the plasma membrane of spines, they were randomly distributed from the synapse. The increase of KCC2 signal in spines with maturation suggests that the main role of KCC2 during perinatal period is spinogenesis. Alternatively, the function of KCC2 in cortical cells is to lower the intracellular Cl^−^ concentration independently from incoming excitatory of inhibitory synapses, rather than the involvement in volume regulation control in response to hypo-osmotic swelling in the hippocampus (Gulyas et al. 2001).

### Functional implications

Our light- and quantitative electron microscopic observations provided support for the view that KCC2 protein is developmentally regulated in postnatal rat brain. We found that the expression is low at birth and is increased in neuron- and layer-specific manner during the development in the cortex. Developmental upregulation of the KCC2 protein has been implicated in the change of GABA signalling in the rat CNS (Ben-Ari et al. 1989; Zhang et al. 1990; Wu et al. 1992; Owens et al. 1996). High [Cl^−^]_i_ have been demonstrated from immature neurons in many brain structures (Owens et al. 1996; Hara et al. 1992; Rohrbough and Spitzer 1996). Therefore, in early postnatal age, GABA_A_-mediated responses were found to be depolarizing (Ben-Ari et al. 1989). A gradual shift toward hyperpolarizing responses is observed around the end of the first postnatal week in the neocortex and 1 week later in the hippocampus (Rivera et al. 1999; Cherubini et al. 1991; Owens et al. 1996; Dammerman et al. 2000; Yamada et al. 2004). This is the period when we found that the adult expression pattern is established in the neocortex. The underlying mechanisms of the high [Cl^−^]_i_ in embryonic neuroblasts and neonatal neurons are still not fully elucidated. Two antagonistically working mechanisms, the presence of an active inward Cl^−^ transport mechanism (NKCC) and the absence of an efficient Cl^−^ extruding system (KCC2), are implicated. Complementary expression pattern of the two cotransporters has been reported (Wang et al. 2002; Clayton et al. 1998). During early postnatal development, as NKCC1 expression decreases KCC2 expression increases. The developmental timing of expression of KCC2 we observed in the neo- and paleocortices very likely accounts for the maturational changes in Cl^−^ homeostasis and GABA function during postnatal life. Cortical neurons lacking KCC2 showed impairment of the intracellular Cl^−^ regulation (Zhu et al. 2005) proving that KCC2 is critical for Cl^−^ homeostasis in mature cortical neurons.

Recent studies highlighted the importance of KCC2 in morphological maturation (Cancedda et al. 2007) and spine density regulation (Fiumelli et al. 2012) of cortical neurons. Interestingly, ion transport function is not necessary for spine genesis, and the important step is the interaction of KCC2 with the dendritic cytoskeleton (Fiumelli et al. 2012; Li et al. 2007; Horn et al. 2010). In line with these recent observations, we found that a large proportion of KCC2 protein is not associated with plasma membrane but rather localized in the cytoplasm. Our finding provides ultrastructural confirmation for the dual role of KCC2: plasma membrane associated KCC2 likely regulates Cl^−^ homeostasis, while in the cytoplasm it plays a role in spino- and synaptogenesis that is independent of the cotransport function of the protein.
